# *Escherichia coli*strains of phylogenetic group B2 and D and bacteriocin production are associated with advanced colorectal neoplasia

**DOI:** 10.1186/s12879-014-0733-7

**Published:** 2014-12-24

**Authors:** Darina Kohoutova, David Smajs, Paula Moravkova, Jiri Cyrany, Monika Moravkova, Miroslava Forstlova, Michal Cihak, Stanislav Rejchrt, Jan Bures

**Affiliations:** 2nd Department of Internal Medicine - Gastroenterology, Charles University in Praha, Faculty of Medicine at Hradec Kralove, University Teaching Hospital, Sokolska 581, Hradec Kralove 500 05 Czech Republic; National Medical Laser Centre, University College London, 67-73 Riding House Street, London, W1W 7EJ United Kingdom; Department of Biology, Masaryk University, Faculty of Medicine, University Campus at Bohunice, Kamenice 753/5, Brno, 625 00 Czech Republic; Department of Clinical Microbiology, Charles University in Praha, Faculty of Medicine at Hradec Kralove, University Teaching Hospital, Sokolska 581, Hradec Kralove, 500 05 Czech Republic; Department of Mathematics, Faculty of Science, University of Hradec Kralove, Jana Koziny 1237, Hradec Kralove, 500 03 Czech Republic

**Keywords:** Escherichia coli, Colicins, Bacteriocins, Colorectal adenoma, Colorectal carcinoma

## Abstract

**Background:**

Colorectal cancer (CRC) is the 3rd most common cancer worldwide and the Czech Republic has the 6th highest incidence of CRC worldwide. Large intestinal microbiota play in its etiopathogenesis important role. Bacteriocins are proteins, produced by bacteria from the *Enterobacteriaceae* family. The aim of our prospective study was to assess the colonization of large intestinal mucosa by *Escherichia coli* strains and to investigate their bacteriocin production.

**Methods:**

A total of 30 consecutive patients with colorectal adenoma, CRA (17 men, 13 women, aged 39–79, mean age 63 ± 9), 30 patients with CRC (23 men, 7 women, aged 38–86, mean age 67 ± 11) and 20 healthy controls (9 men, 11 women, age 23–84, mean age 55 ± 15) were enrolled into prospective study. Mucosal biopsies were taken in the caecum, transverse colon and rectum during pancolonoscopy. Microbiological culture, isolation and identification of bacteria followed. Bacteriocin production was assessed by growth inhibition of indicator strains *E. coli* K12-Row*, E. coli* C6 (phi), and *Shigella sonnei* 17. Identification of bacteriocin-encoding determinants and *E. coli* phylogroups was performed using PCR methods.

**Results:**

A total of 622 strains were isolated and further investigated. A significantly higher frequency of simultaneous production of colicins and microcins was revealed in the group of patients with CRC, when compared to patients with CRA, p = 0.031. A significantly higher frequency of *E. coli* phylogroup D was found in patients with CRC, when compared to controls, p = 0.044. A significantly higher prevalence of bacteriocinogeny was confirmed in patients with advanced adenoma when compared to patients with non-advanced adenoma, p = 0.010. Increasing bacteriocinogeny was associated with an increasing stage of CRC (assessed according to TNM classification). Either *E. coli* phylogroup B2 or *E. coli* phylogroup D were isolated in biopsies of patients with right-sided CRC. A statistically higher incidence of *E. coli* phylogroup B2 was found in patients with right-sided CRC when compared to patients with left-sided CRC, p = 0.028.

**Conclusions:**

Large intestinal mucosa of patients with more advanced colorectal neoplasia is colonized with more virulent strains of *E. coli* and higher production of bacteriocins is observed in these patients when compared to those with less advanced colorectal neoplasia.

**Electronic supplementary material:**

The online version of this article (doi:10.1186/s12879-014-0733-7) contains supplementary material, which is available to authorized users.

## Background

Colorectal cancer (CRC) is the 3rd most common cancer having been diagnosed in nearly 1.4 million people worldwide in 2012. The Czech Republic had the 6th highest incidence of CRC worldwide and the age-standardized rate per 100 000 was 38.9 in 2012 [1]. Despite previous efforts, the etiopathogenesis of CRC has not been fully clarified yet and prevention does not exist. Large intestinal microbiota, undoubtedly, play an important role in the CRC pathogenesis [2],[3].

Even though a considerable effort was put into the development of new antineoplastic therapeutic strategies, severe side effects and resistance of colon cancer cells towards the therapy remain limiting hurdles [4],[5].

Bacteriocins possess antimicrobial [6], proapoptotic [7], and probiotic properties [8]. Although the exact role of bacteriocins is not known on both bacterial population level and at the level of their interaction with the eukaryotic host, several bacteriocins posess antineoplastic properties according to in vitro studies [9],[10]. Colicins, microcins, pyocins and pediocins belong to the bacteriocins with a reported antineoplastic activity [11].

Bacteriocins are produced by *Escherichia coli* strains and related bacteria from the *Enterobacteriaceae* family [12],[13].

The aim of our prospective study was to assess whether there are differences in *E. coli* colonization of the large intestinal mucosa between the groups tested and to investigate bacteriocin production in the patients with colorectal adenoma (CRA) and CRC.

## Methods

A total of 30 consecutive patients with CRA (17 men, 13 women, aged 39–79, mean age 63 ± 9), 30 patients with CRC (23 men, 7 women, aged 38–86, mean age 67 ± 11) and 20 healthy controls (population with average risk for CRC with normal endoscopic findings and with negative history of CRA, CRC or inflammatory bowel disease; 9 men, 11 women, age 23–84, mean age 55 ± 15) were enrolled into the prospective study.

There were 6 patients with non-advanced (N-A) CRA and 24 patients with advanced (A) CRA. Advanced CRA is either an adenoma with low grade dysplasia larger than 10 mm and/or adenoma with high grade dysplasia of any size and/or adenoma with villous component found on histology. The group with CRC included 4 patients with right-sided CRC and 26 patients with left-sided CRC. The border between the “left” and the “right” colon was at the lineal flexure of the colon.

Individuals enrolled into the study were invited for the diagnostic and/or therapeutic pancolonoscopy. The usual bowel preparation was either polyethylene glycol or sodium phosphate solution. Pancolonoscopy was performed in a standard manner under conscious sedation in all the subjects. Video-colonoscopes Olympus 160 series (Olympus Corp., Tokyo, Japan) were used after previous high-level disinfection for each particular investigation (ETD2 and ETD3 disinfectors Olympus). Mucosal biopsies were taken from the caecum, transverse colon and the rectum during the procedure in all the patients/healthy controls (90 biopsies in patients with CRA, 90 biopsies in patients with CRC and 60 biopsies in the control group). Sterile biopsy forceps (Olympus) were used for every single biopsy. In our previous study we verified that the inner space of the forceps remained sterile despite the passage of the accessory through the working channel of the endoscope (data not shown). Each biopsy specimen of the colonic mucosa was immediately inserted into a transport liver-enriched broth. Standard primary cultures were inoculated on the blood and MacConkey agars (at 37°C for 24 hours); this was followed by a standard clone isolation. Up to 7 different colonies of the coliform bacteria were isolated from each sample (on the blood, MacConkey and deoxycholate agars). Particular bacteria were precisely identified by the Vitek2 system (BioMérieux, Marcy l’Etoile, France) and susceptibility of the strains to the antibiotics was assessed. Together, 622 isolates were identified: 221 in the group of patients with CRA, 151 in the healthy controls and 250 in the patients with CRC. All the bacterial strains were frozen in cryotube vials at −80°C appropriately.

Bacteriocinogeny (bacteriocin production) of each strain, bacteriocinogenotypisation (determination of bacteriocin type) and *E. coli* phylogroups were further investigated. Frozen bacterial strains were streaked for single colonies and one single colony was used for inoculation of liquid TY medium containing tryptone (Hi-Media, Mumbai, India) 8 g/L, yeast extract (Hi-Media) 5 g/L, and sodium chloride 5 g/L. The agar plates (containing the 1.5% TY agar, w/v) were subsequently inoculated by needle stab with fresh broth cultures and the plates were incubated at 37°C for 48 hours. The bacteria were killed using chloroform vapours for 30 minutes. Each plate was overlaid with a thin layer of a soft agar (0.7% TY agar, w/v) containing 10^7^ cells/mL of an indicator strain. The plates were then incubated at 37°C overnight. With the exception of TY agar plates, bacteriocin production was tested on a relatively unenriched agar containing a Difco™nutrient broth (Difco Laboratories, Sparks, MD) 8 g/L, sodium chloride 5 g/L, and 1.5% (w/v) agar. Indicator strains of *E. coli* K12-Row*, E. coli* C6 (phi) and *Shigella sonnei* 17 were used for the assessment of bacteriocin production (a zone of growth inhibition is present around the strain tested, if the indicator strain is susceptible to the bacteriocin produced by the strain tested). All these indicator strains were from our in-house collection of strains. Bacteriocin production was also confirmed by PCR methods. Bacteriocinogenotypisation and determination of *E. coli* phylogroups were investigated by PCR methods using specific primers for the detection of colicin, microcin genes (see Tables 1 and 2 for details) and genes specific for each *E. coli* phylogroup [14]. Altogether, 23 individual colicin types (colicins A, B, D, E1-E9, Ia, Ib, Js, K, L, M, N, S4, U, Y, 5/10) and 8 microcin types (mB17, mC7, mE492, mH47, mJ25, mL, mM, mV) were tested in this study [15]; see Additional file 1 for details.

**Table 1:** **Primers used for the detection of colicin genes**

Colicin	Primer	Sequention of the primer	Lenght of the PCR product
**A**	ColA-F	cgtggggaaaagtcatcatc	475
	ColA-R	gctttgctctttcctgatgc	
**B**	colicinB-F	aagaaaatgacgagaagacg	492
	colicinB-R	gaaagaccaaaggctataagg	
**D**	ColD-F	ctggactgctgctggtgata	420
	ColD-R	gaaggtgcgcctactactgc	
**E1**	colicinE1-F	tgtggcatcgggcgagaata	649
	colicinE1-R	ctgcttcctgaaaagcctttt	
**E1-1**	cea2F	ggtggaactggaggtagcaa	357
	ceaR	acgtcgttgttctgcttcct	
**E2**	ColE2-F	tgatgctgctgcaaaagag	409
	ColE2-R	ttcaaagcgttccctaccac	
**E3**	ColE3-F	taagcaggctgcatttgatg	413
	ColE3-R	tcggatctggacctttcaac	
**E4**	ColE4-F	gaaggctgcatttgatgct	409
	ColE4-R	cggatccggacctttaattt	
**E5**	ColE3-F	taagcaggctgcatttgatg	430
	ColE5-R	ttgaattctcgaatcgtcca	
**E6**	ColE6-F	accgaacgtccaggtgtt	399
	ColE6-R	tttagcctgtcgctcctgat	
**E7**	ColE7-F	gcattctgccatctgaaat	431
	ColE7-R	cttctgcccactttctttcg	
**E8**	ColE3-F	taagcaggctgcatttgatg	449
	ColE8-R	gactgattggcttgtcgtga	
**E9**	ColE3-F	taagcaggctgcatttgatg	418
	ColE9-R	gacttttctccctccgacct	
**Ia**	ColIa-F	gcatgcaaatgacgctctta	473
	ColIa-R	gaggacgccagttctctgtc	
**Ib**	ColIb-F	aacgagtgggtcgatgattc	464
	ColIb-R	ccttttctgcgctcgtattc	
**Js**	ColJs-F	tcaaaatgtttgggctcctc	254
	ColJs-R	taatctgccctgtcccactg	
**K**	ColK-F	cagaggtcgctgaacatgaa	469
	ColK-R	tccgctaaatcctgagcaat	
**L**	Col28b(L)-F	tgcatattgaaagcgtcagc	449
	Col28b(L)-R	caggttatcccctctcacca	
**M**	ColM-F	gcttaccacttcgcaaaacc	429
	ColM-R	gagcgactctccgataatgc	
**N**	ColN-F	agcttggcgagtatcttgga	401
	ColN-R	caacacagccccgaataaac	
**S4**	ColS4-F	tatatggcccaactgctggt	456
	ColS4-R	cgtaaggacggacacctgtt	
**U**	ColU-F	tgattgctgcgagaaaaatg	485
	ColU-R	tctgacagcctctccctgtt	
**Colicin**	**Primer**	**Sequention of the primer**	**Lenght of the PCR product**
**Y**	ColY-F	gcaggcagaaaagaacaagg	477
	ColY-R	cggacgttatttgccttcat	
**5**	Col5-F	cattggcaaaagcgaaatct	443
	Col5-R	tgcaactctggaaacaatcg	
**10**	Col10-F	ggttaccggatttcctggat	448
	Col10-R	ttctagatgcttggcccact	
**Fy**	ColFy-Fa	aaattaagcggtgccattgac	580
	ColFy-Fa	ttctaattgcgccagacctt

**Table 2:** **Primers used for the detection of microcin genes**

Microcin	Primer	Sequention of the primer	Lenght of the PCR product
**B17**	mcc B17-F	tcacgccagtctccattaggtgttggcatt	135
	mcc B17-R	ttccgccgctgccaccgtttccaccactac	
**C7**	mcc C7-F	cgttcaactgttgcaatgct	134
	mcc C7-R	agttgaggggcgtgtaattg	
**E492**	mcc E492-F	gtctctcctgcaccaaaagc	291
	mcc E492-R	ttttcagtcatggcgttctg	
**H47**	mcc H47-F	cactttcatcccttcggattg	227
	mcc H47-R	agctgaagtcgctggcgcacctcc	
**J25**	mcc J25-F	tcagccatagaaagatataggtgtaccaat	175
	mcc J25-R	tgattaagcattttcattttaataaagtgt	
**L**	mcc L-F	ggtaaatgatatatgagagaaataacgtta	233
	mcc L-R	tttcgctgagttggaatttcctgctgcatc	
**V**	mcc V-F	cacacacaaaacgggagctgtt	680
	mcc V-R	tttcgctgagttggaatttcctgctgcatc	
**M**	micM-4-F	cgtttattagcccgggattt	166
	micM-4-R	gcagacgaagaggcacttg	

Data obtained were treated statistically by means of descriptive statistics, non-paired t-test and Fisher’s exact test using Statistica software. The differences in bacteriocinogeny, colicinogeny and microcinogeny between the groups tested were assessed by non-paired t-test (as this was referred to the number of biopsies taken in each group: 90 vs 90 vs 60). Fisher t-test was used for investigation of differences in production of each bacteriocin between the groups tested (as this was reffered to the number of individuals in each group: 30 vs 30 vs 20 and the numbers were small).

All individuals enrolled into the study were adult persons. Participants were adequately informed and a written informed consent for participation in the study including agreement with the data and information to be published in an anonymous way was obtained from all the enrolled subjects. The project was approved by the Joint Ethical Committee (Charles University in Praha, Faculty of Medicine at Hradec Kralove & University Teaching Hospital Hradec Kralove). For all data obtained, all personal identification information was removed in compliance with the Czech laws for protection of confidentiality.

## Results

Microbiological culture was performed on 240 mucosal biopsies taken from 60 patients (30 patients with CRC, 30 patients with CRA) and 20 healthy controls. Together 622 strains were isolated and futher investigated. There were no statistically significant differences in the frequency of strains belonging to the *Enterobacteriaceae* family between our groups investigated. A significantly higher frequency of *Escherichia coli* strains was found in the patients with CRC, when compared to the healthy controls, p < 0.001; see Table 3 for details.

**Table 3:** **Characteristics of large intestinal microbiota, bacteriocins and** ***E*** **.** ***coli*** **phylogroups in each investigated group of patients/controls**

	Adenoma	Carcinoma	Controls
Number of biopsies with ≥1 strain belonging to *Enterobacteriaceae* family per all biopsies taken in each group	89/90 (99%)	89/90 (99%)	60/60 (100%)
Bacteriocinogeny	58/89 (65%)	61/89 (69%)	39/60 (65%)
Colicinogeny	31/89 (35%)	40/89 (45%)	24/60 (40%)
Microcinogeny	46/89 (52%)	53/89 (60%)	36/60 (60%)
Simultaneous colicinogeny and microcinogeny	19/89 (21%)	32/89 (36%)	21/60 (35%)
Frequency of *E. coli* from *Enterobacteriaceae* strains	77/89 (87%)	87/89 (98%)	48/60 (80%)
*E. coli* - phylogroup A	31/77 (40%)	30/87 (34%)	21/48 (44%)
*E. coli* - phylogroup B1	19/77 (25%)	18/87 (21%)	12/48 (25%)
*E. coli* - phylogroup B2	35/77 (45%)	41/87 (47%)	24/48 (50%)
*E. coli* - phylogroup D	16/77 (21%)	24/87 (28%)	6/48 (13%)

The details refer to the numbers of biopsies taken in each group of individuals.

There was no difference in colicinogeny and microcinogeny between the groups tested. A significantly higher frequency of simultaneous production of colicins and microcins was revealed in the group of patients with CRC, when compared to patients with CRA, p = 0.031; see Table 3 for details. A trend towards higher co-production of colicins and microcins in the healthy controls, when compared to the patients with CRA, was revealed, p = 0.065; see Table 3 for details. A statistically significant difference in bacteriocinogeny was found between patients with non-advanced (N-A) and patients with advanced (A) CRA: N-A: 7/18 (39%), A: 51/71 (72%); p = 0.010.

Colicin Ia was the most commonly synthetised colicin in all the groups tested. It was followed by the colicin Ib in the patients with CRA and in the healthy controls. In the patients with CRC, the colicin M belongs to the 2nd most frequently synthetised colicin after the colicin Ia. Colicin M was produced by 4% of patients with CRA, 10% of healthy individuals and 21% of patients with CRC. There was a statistically significant difference in production of colicin M between the patients with CRA and CRC (p = 0.001) and a trend towards higher production of colicin M in the patients with CRC, when compared to the healthy controls, was identified; p = 0.070. Microcin mH47 was the most frequently produced microcin in all the groups tested. In the patients with CRA, it was followed by microcin mV. Synthesis of microcin mM was the second most common after microcin mH47 in the patients with CRC and in the healthy individuals.

The lowest incidence of *E. coli* phylogroup A and B1 were found in the group of the patients with CRC. A significantly higher frequency of *E. coli* phylogroup D was found in the group of patients with CRC, when compared to the healthy controls, p = 0.044. Similar frequency of *E. coli* phylogroup B2 was found in all the groups tested; see Table 3 for details. A trend towards lower frequency of *E. coli* phylogroup B2 was revealed in the group of patients with N-A CRA, when compared to the patients with A CRA: N-A: 4/17 (24%), A: 31/60 (52%); p = 0.054. Either *E. coli* phylogroup B2 or *E. coli* phylogroup D were isolated in all biopsies, which were taken in the patients with right-sided CRC. A statistically significant difference in the frequency of *E. coli* phylogroup B2 was revealed between patients with right-sided CRC and patients with left-sided CRC: right-sided: 9/12 (75%), left-sided: 32/78 (41%); p = 0.028.

Increasing bacteriocinogeny, colicinogeny, microcinogeny and colicinogeny & microcinogeny was associated with an increasing stage of CRC (assessed according to the TNM classification), see Table 4 for details. A statistically significant difference was found in microcinogeny between the stage 1 and stage 4: p = 0.038.

**Table 4:** **Characterictics of bacteriocinogeny in patients with CRC**

Staging (TNM classification)	I	II	III	IV
Bactericinogenic strains	6/12 (50%)	10/12 (83%)	26/38 (68%)	6/6 (100%)
Colicinogenic strains	3/12 (25%)	4/12 (33%)	20/38 (53%)	4/6 (67%)
Microcinogenic strains	5/12 (42%)	8/12 (67%)	24/38 (63%)	6/6 (100%)
Strains producing colicin and microcin	2/12 (17%)	2/12 (17%)	18/38 (47%)	4/6 (67%)

Association between increasing bacteriocinogeny, colicinogeny, microcinogeny, colicinogeny & microcinogeny with an increasing stage of CRC. A statistically significant difference was found in microcinogeny between the stage 1 and stage 4: p = 0.038.

## Discussion

Large intestinal microbiota, undoubtedly, play an important role in the pathogenesis of CRC [2],[3]. *E. coli* can be classified into 3 major groups: commensal strains, intestinal pathogenic strains and extraintestinal pathogenic strains [16],[17]. Phylogenetic diversity of *E. coli* exists within each subgroup [16]. Four phylogroups of *E. coli* are well-known. Commensal strains usually belong to the A or B1 phylogenetic group; pathogenic strains usually belong to B2 or D phylogroup of *E. coli* and they possess more virulence factors when compared to the commensal strains [16],[18],[19]. Among our groups tested, the lowest incidence of *E. coli* phylogroup A and B1 and the highest frequency of *E. coli* phylogroup D was revealed in the patients with CRC. This finding shows that the large intestinal mucosa of the patients with CRC is colonized with more virulent *E. coli* strains. We also confirmed in our study, that large intestinal mucosa of patients with CRC is inhabited with a significantly higher proportion of *Escherichia coli* strains when compared to the healthy controls.

*E. coli* strains of the B2 phylogenetic group harbour the “pks” genomic island, which encodes production of a non-ribosomally synthetised polyketide-peptide genotoxin, called colibactin [20]. A study carried out by Cuevas-Ramos *et al.* documented that even a single and short exposure of mammalian epithelial cells to “pks” positive *E. coli* strains at low infectious doses induced DNA-double strand breaks, signs of incomplete DNA repair during the cell division which finally lead to the chromosome aberrations [21]. Infection caused by “pks” positive *E. coli* strains affects the host immune response and is accompanied by the production of oxygen species, pro-inflammatory cytokines and protease secretion. These mediators are able to trigger DNA-double strand breaks [22],[23]. Nowrouzian *et al.* substantiated the contribution of “pks” island to long-term gut-colonization of *E. coli* B2 strains [20]. Association of colonic mucosa of patients with CRC and enterotoxigenic *E. coli* belonging to the B2 phylogroup, which produces toxins called cyclomodulins, has been also highlighted recently [24]. Despite all recent efforts, the precise etiopathogenesis of right-sided colorectal cancer has not yet been clarified [25]. The high occurrence of the mutagenic *E. coli* B2 phylogroup in our patients with right-sided colorectal cancer could elucidate the pathogenesis and difference in the pathways of cancer development when compared to the patients with left-sided colorectal cancer. These conclusions are in agreement with those of Cuevas-Ramos, who assumed that colon colonization with “pks” positive *E. coli* strains contributes to the development of sporadic CRC [21]. A study carried out by Arthur *et al.* also confirmed that the deletion of the “pks” genotoxic island from *E. coli* NC101 decreased intestinal tumor multiplicity and invasion in experimental mice, without altering intestinal inflammation [26]. A higher frequency of *E. coli* phylogroup B2 in the patients with advanced CRA when compared to the patients with non-advanced CRA in our study confirms the anticipated involvement of “pks” positive *E. coli* strains in the pathogenesis of CRC.

The antineoplastic effect of colicins has been described in in vitro studies: colicin E3 is able to inhibit proliferation of leukemic cells [27],[28] and shows antineoplastic effect towards HeLa cells, epithelial cells derived from the cervix [29]. The cytotoxic effect of colicins was also proven against breast cancer cells [30]. Farkas-Himsley substantiated in an experimental study, that the cells of colorectal adenocarcinoma were more sensitive to bacteriocins when compared to normal cells of large bowel mucosa [31]. Bures *et al.* studied colicin production in the patients with CRC and found a significantly higher colicinogeny in the healthy controls when compared to the patients with CRC [12]. This was not confirmed in another study performed by Smarda *et al.* [32]. Our current study is in agreement with that of Smarda *et al.* in that we have not found a difference in bacteriocinogeny between the patients with CRC and the healthy controls. However we found a significantly higher simultaneous production of colicins and microcins in the patients with CRC when compared to the patients with CRA. Patients with advanced CRA had also a statistically higher bacteriocinogeny when compared to those with non-advanced CRA. Increasing bacteriocinogeny associated with an increasing stage of CRC (when assessed according to the TNM classification) highlights the previous findings: there is an increasing bacteriocin production from non-advanced CRA to advanced CRA and from non-advanced CRC to advanced CRC. Large intestinal mucosa of the patients with more advanced colorectal neoplasia is also colonized with more virulent strains of *Escherichia coli.*

We presume that the intermicrobial competition (for nutrition, etc.) in the large bowel of healthy subjects can be conveyed as a high simultaneous production of microcins and colicins. We may hypothetize that if the production of antimicrobial, antiapoptotic and potentially antineoplastic bacteriocins decreases in the healthy individuals, this could co-iniciate the development of a non-advanced colorectal neoplasia. With the growth, development and progression of a non-advanced neoplasia into an advanced one, higher production of bacteriocins is again identified and also more virulent *E. coli* strains are present. We assume, that this situation could be beneficial for the host and might help the macroorganism in combating the colorectal cancer. Our hypothesis needs furher investigation especially in the field of potential antineoplastic properties of the bacteriocins and clarification of their interaction with the eukaryotic host.

## Conclusions

Large intestinal mucosa of the patients with more advanced colorectal neoplasia is colonized with more virulent strains of *Escherichia coli* and a higher production of bacteriocins is observed in these patients when compared to those with less advanced colorectal neoplasia.

### Availability of supporting data

The data sets supporting the results of this article are included within the article and its additional file.

## Additional file

## Electronic supplementary material

Additional file 1: Sheet 1: information regarding patients with colorectal cancer (CRC). Sheet 2: information regarding patients with colorectal adenoma (CRA). Sheet 3: information regarding healthy controls (Controls). (XLS 60 KB)
